# Affective Temperament Traits and Age-Predicted Recreational Cannabis Use in Medical Students: A Cross-Sectional Study

**DOI:** 10.3390/ijerph17134836

**Published:** 2020-07-05

**Authors:** Carmenrita Infortuna, Steven Silvestro, Keith Crenshaw, Maria Rosaria Anna Muscatello, Antonio Bruno, Rocco Antonio Zoccali, Eileen Chusid, Jordan Intrator, Zhiyong Han, Fortunato Battaglia

**Affiliations:** 1 Department of Biomedical and Dental Sciences and Morphofunctional Imaging, Policlinico Universitario, University of Messina, 98124 Messina, Italy; carmen.infortuna@gmail.com (C.I.); mmuscatello@unime.it (M.R.A.M.); antonio.bruno@unime.it (A.B.); rocco.zoccali@unime.it (R.A.Z.); 2 Department of Pre-Clinical Sciences, New York College of Podiatric Medicine, New York, NY 10035, USA; SSilvestro2022@nycpm.edu (S.S.); KCrenshaw2022@nycpm.edu (K.C.); echusid@nycpm.edu (E.C.); 3 Department of Medical Sciences, Neurology and Psychiatry, Hackensack Meridian School of Medicine at Seton Hall University, Nutley, New Jersey, NJ 07110, USA; jordan.intrator@shu.edu (J.I.); zhiyong.han@shu.edu (Z.H.)

**Keywords:** temperament, cannabis, medical students, cyclothymic, irritable

## Abstract

The use of cannabis among college students is increasing. Cannabis abuse has been proposed to be associated with personality dimensions. However, there are currently no known studies on the relationship of temperament traits and recreational cannabis use among college students. This is a cross-sectional study that investigated 328 students at a Podiatric Medical College. We evaluated the association between temperament and recreational cannabis use by the students. Temperament was investigated using the Memphis, Pisa, Paris and San Diego Auto- Questionnaire (TEMPS-A (short version)). Additionally, we assessed demographics variables and perceived stress in the context of cannabis use, and analyzed the findings using logistic regression. The prevalence of recreational cannabis use was 8.45%. Recreational cannabis use among these students was highly associated with irritable and cyclothymic temperament traits. There was no association between recreational cannabis use and perceived stress, and demographic variables or other substance use. Furthermore, logistic regression analysis indicated that higher scores in cyclothymic or irritable temperament traits are significant predictors for recreational cannabis use. Our study has identified key temperament traits, with a strong association with recreational use of cannabis of the studied student population. Our findings are useful in designing screening and educational strategies directed towards increasing the wellbeing of medical students.

## 1. Background

Cannabis is now the third most widely used psychoactive substance, behind nicotine-containing products and alcohol [1]. About 30% of undergraduate college students reported past-year cannabis use, a rate similar to that of cannabis use by non-college individuals of the same age group [2,3]. While one might expect that the prevalence would be high during late adolescence and early adulthood, there are studies indicating that the recreational use of cannabis is not limited to this demographic. For instance, a 2018 systematic review on cannabis use by medical students reported a past-month use of cannabis by 8.8% of students [4]. It has been proposed that the rate of cannabis use among medical students is associated with exposure to diverse stressors and an attempt to prevent burnout [5]. This proposition stems from the findings that show the general public uses cannabis as a way of self-treatment of stress, anxiety, and depression [6]. However, more research is needed to investigate whether this is true with podiatric medical students. There are noticeable parallels between the curricula of podiatric medicine, osteopathic medicine, and allopathic medicine, in that each curriculum is comprised of two years of pre-clinical science training followed by two years of clinical science training, which place similar demands on students. Therefore, the results from examining podiatric medical students could be generalized to osteopathic medical students and allopathic medical students.

In contrast to environmental factors as possible contributing factors for cannabis use, personal factors, such as temperament, may be associated with a tendency of substance use in students as well as others. Temperament is a feature of a person’s personality that is present early in life, is stable over time, and is possibly partially determined by biological determinants [7]. It has been well demonstrated that certain temperament traits are linked to an increased rate of substance use in general, and life-time substance abuse; however, less is known about the link between temperament and cannabis use [8]. Furthermore, no studies have investigated whether there is an association between specific temperament traits and recreational cannabis use among medical students. This is an important issue because, with the liberalization of laws and regulations related to cannabis in the US, an increase in cannabis use among more Americans is expected. Thus, more information is needed to characterize the risk factors associated with cannabis use, both in the public and in unique sub-populations.

The primary objectives of the study were: (1) to investigate the prevalence of past month recreational cannabis use in podiatric medical students; (2) to identify the association of cannabis use with emotional temperament traits and perceived stress. We hypothesize that, since certain temperament traits and perceived stress are associated with substance abuse, these same factors will be associated with the past month’s recreational cannabis use by podiatric medical students.

## 2. Methods

This cross-sectional study was performed in a convenience sample at a Medical College located in New York City. A total of consenting 328 students, from academic years one through four, were administered a survey (May–June 2019). Of these participants, 297 completed and submitted the survey documents. Surveys were administered using paper copies. Before they were given the survey documents, students were given a brief presentation informing them of the study purpose, study associated confidentiality measures, instructions on how to correctly mark options, and providing them reassurance that participation is voluntary. All study data received from participants were de-identified. The survey had a statement of written consent (“Do you agree to the above terms? By selecting Yes, you consent that you are willing to answer the questions in this survey and that you authorize us to use the data for research purpose’). The participants were requested to place questionnaire documents into a blank envelope, and to place it into an empty box. The study was approved by the local Institutional Review Board (New York College of Podiatric Medicine).

### 2.1. Measures

The main goal of this study was to investigate the prevalence of recreational cannabis use among podiatric medical students, and whether it associates with temperament traits and perceived stress. Section 1 of the survey gathered information regarding demographics of the participants (age, gender, medical school year, marital status, parental status) and grade point average (GPA).

The Memphis, Pisa, Paris and San Diego Auto-Questionnaire (TEMPS-A) short version was used to assess affective temperament traits [9]. The scale allows the investigation of five temperament traits: cyclothymic, depressive, irritable, hyperthymic, anxious. The scale has been translated into many languages, and has been widely used and validated [9,10]. The Cronbach’s alpha in this study was 0.812.

The Perceived Stress Scale-10 (PSS-10) is a tool to estimate the degree to which the participants evaluated life events as stressful during the past month [11]. The PSS-10 scale contains 10 items with a 5-points Likert scale. A higher score indicates high perception of stress during the last month. This scale has been used extensively, and has good psychometric properties [12]. The Cronbach’s alpha in this study was 0.831.

Recreational cannabis use was determined by asking the participants if they used cannabis during the last month: “During the past month, have you intentionally self-administered cannabis, cannabis-products, or ingredients derived from cannabis by methods including, but not limited to, smoking, eating, or vaporizing? (yes/no)”.

### 2.2. Statistical Analysis

The sample size was calculated using G*Power software [13]. We first calculated frequencies, prevalence of cannabis use, subjective rating of perceived stress, and affective temperaments (mean ± SD). The comparison of nominal variables was made using the χ^2^ test. ANOVA was used to compare between-group difference of continuous variables.

A multivariate logistic regression model including all assessed variables was used to determine the independent factors associated with recreational, past month cannabis use. SPSS Statistics for Windows, Version 23 (IBM Corporation, Armonk, NY, USA) was used for all analyses. For all calculations, the alpha level was set to <0.05.

## 3. Results

The completed survey response rate was 90.2% (296 of 328 students). The demographic variables and GPA are reported in Table 1. The mean age of the participants was 26.9 ± 2.3 years (183 students ages ≤ 25 and 113 students ages ≥ 26). A total of 50% of the students self-identified as Caucasian, 5% as African American, 35% as Asian, 7% as Hispanic and 2% as others. There was an equal representation of male and female students (Female, *N* = 153, 51.69%; Male, *N* = 143, 48.31%). Of the respondents, 81 (27.20%) were from the first-year class, 74 (24.90%) from the second-year class, 64 (21.50%) from the third-year class, and 77 (25.90%) from the fourth-year class. Most of the students (209, 70.61%) were single and had no children (275, 92.91%). A total of 12 (4.05%) students had a GPA below 2.5, 67 (22.64%) had a GPA between 2.5 and 2.9, 121 (40.88%) had a GPA between 3.0 and 3.4 GPA, and 96 (32.43%) students had a GPA above 3.5. Twenty-five students (8.45%) used cannabis in the past month.

There was a gender difference in recreational cannabis use, with a higher prevalence in males (*X^2^* = 7.2, *p* = 0.007). There was no difference in the frequency of recreational cannabis use when other variables were analyzed: Age, *X^2^* = 0.486, *p* = 0.486, Academic year, *X^2^* = 3.783, *p* = 0.052; Marital status, *X^2^* = 4.117, *p* = 0.043; Parental status, *X^2^* = 0.045, *p* = 0.832; GPA, *X^2^* = 0.174, *p* = 0.676, PSS-10, *X^2^* = 0.001, *p* = 0.972.

We then evaluated the between-group differences among cannabis users and non-users, using the mean score of the PSS-10 and the four domains of the TEMPS-A. Significant differences were found only for the cyclothymic and irritable traits (PSS-10: non-users 18.24 ± 0.43, users 18 ± 1.48, F_(1, 295)_ = 0.02, *p* = 0.87; Cyclothymic: non-users 3.42 ± 0.15, users 4.48 ± 0.43, F_(1, 295)_ = 4.12, *p* = 0.04; Depressive: non-users 3.82 ± 0.18, users 3.36 ± 0.48, F_(1, 295)_ = 0.54, *p* = 0.46; Irritable: non-users 2.95 ± 0.14, users 4.84 ± 0.43, F_(1, 295)_ = 34.1, *p* < 0.0001; Hyperthymic: non-users 3.72 ± 0.11, users 3.3 ± 0.39, F_(1, 295)_ = 3.28, *p* = 0.07; Anxious: non-users 2.9 ± 0.09, users 3.2 ± 0.41, F_(1, 295)_ = 0.81, *p* = 0.36) Table 2.

A logistic regression was performed to ascertain the effects of age, gender, marital status, GPA, perceived stress, and affective temperament traits on the likelihood that participants were cannabis users in the past month. The logistic regression model was statistically significant, χ^2^(8) = 32.87, *p* < 0.0001. The model explained 51.5% (Nagelkerke R2) of the variance in recreational, past-month cannabis use, and correctly classified 91.5% of the cases. Males were 0.22 times more likely to be users than females. The cyclothymic and irritable traits were associated with an increased likelihood of being a past-month recreational cannabis user (Table 3).

## 4. Discussion

Evidence from current literature on substance abuse prompted us to hypothesize that both temperament traits and perceived stress would be associated with recreational cannabis use in podiatric medical students. The association between temperament traits and past-month cannabis use is supported by our results, whereas we did not find an association between perceived stress and past-month cannabis use.

In our study, 8.45% of the students used cannabis in the last month. This result is in agreement with the findings of previously published studies. For instance, in a recent meta-analysis of 25 studies that had investigated past-month cannabis use in medical students, Papazisis et al. found an estimated prevalence of 8.8% [4]. Although published results are rather heterogeneous, when considering studies assessing larger cohorts, the data nevertheless seems in keeping with our results [14,15,16,17,18,19,20,21].

We found that male students had higher odds of being past-month users. This is similar to studies by others showing a higher prevalence of cannabis use among males medical students [22]. Gender differences in substance use disorders are well-documented [23], although the mechanisms underlying this association are still to be determined. When gender differences are assessed, males appear at greater risk than females for having substance use problems [24], and they are more likely to smoke marijuana recreationally [25]. Future studies are needed to address the interplay between gender and biological and social characteristics influencing medical student behaviors.

This study showed that higher scores in the cyclothymic and irritable temperaments domains are associated with higher odds of cannabis use in the past month by podiatric medical students. Temperament refers to traits that are present at birth and maintained throughout life, and regulate the behavioral response to the environmental stimuli. Previous studies of podiatric medical students have shown that temperament is related to substance abuse in an individual’s life [8] and to non-medical use of prescription stimulants (NMUPS) in [12]. The cyclothymic trait is characterized by emotional instability, swinging mood, and abnormal emotionality, while the irritable temperament shows a predisposition for abnormal reactivity and self-regulation to negative events, hostility, and aggression. Our results confirm and expand previous works which indicate that cyclothymic and irritable temperaments are associated with health-risk behaviors (alcohol, nicotine and cannabis use) among undergraduate students [26]. Since both cyclothymic and irritable temperament traits are stable and have been associated with substance use disorders [10,27,28], our results might have a pivotal importance in planning educational strategies which aim to change a trajectory that potentially leads to the development of addiction in high-risk podiatric medical students.

Our study has some limitations. This is a cross-sectional study; hence, we can only discuss associations between variables, and due to the study design, sample size, and the selected variables, we cannot exclude a volunteer bias. Longitudinal studies are needed to address causality. In addition, self-reported measures of cannabis use have the potential of inaccurate reporting. Future studies should investigate additional psychological variables and health-risk behaviors. Furthermore, as the podiatric medical curriculum is slightly different than other medical colleges, future studies including students from different medical programs should be planned to generalize the results.

## 5. Conclusions

The current body of literature lacks information regarding the driving factors of recreational cannabis use among podiatric medical students. As the male gender and the irritable and cyclothymic temperament traits were strongly associated with recreational cannabis use in our study, these results might prompt educators in both podiatric medical colleges, as well as in other medical schools, to implement screening and prevention strategies to promote mental health and a healthy, caring medical school climate.

## Figures and Tables

**Table 1:** Demographic characteristics, grade point average (GPA), and prevalence of past month cannabis use of the sampled population.

*N* = 296		*N*	%
**Gender**	Male	143	48.31%
	Female	153	51.69%
**Age**	≤25	183	62%
	≥26	113	38%
**Race**	Caucasian	147	50%
	African American	16	5%
	Hispanic	21	7%
	Asian	105	35%
	Others	7	2%
**Academic Year**	Year 1	81	27.20%
	Year 2	74	24.90%
	Year 3	64	21.50%
	Year 4	77	25.90%
**Marital status**	Single	209	70.61%
	Partnered	87	29.39%
**Parental Status**	Children	21	7.09%
	No Children	275	92.91%
**GPA**	<2.5	12	4.05%
	2.5–2.9	67	22.64%
	3–3.4	121	40.88%
	>3.5	96	32.43%
**Cannabis Recreational Use**		25	8.45%

**Table 2:** Between-groups differences among cannabis users and non-users in mean score of the perceived stress scale 10 (PSS-10) and the five domains of the Memphis, Pisa, Paris and San Diego Auto-Questionnaire (TEMPS-A) scale.

Variables	Cannabis Users	Cannabis Non-Users	*p*
Cyclothymic	4.35	3.42	0.0031 **
Depressive	3.42	3.82	0.8127
Irritable	6.62	2.96	<0.0001 **
Hyperthymic	2.96	3.73	0.2664
Anxious	3.15	2.9	0.5366
PSS-10	17.62	18.24	0.9718

Data are Mean ± SD. ** *p* < 0.001.

**Table 3:** Multinomial Logistic Regression Predicting past month cannabis use in podiatric medical students. The model includes demographic variables, GPA, PSS-10, and Temps-A temperament dimensions.

Variables	B	S.E.	Wald	df	Sig.	Exp (B)
Age (1)	−0.073	1.349	0.003	1	0.957	0.93
Gender (1)	−1.494	0.692	4.653	1	0.031 *	0.225
Year			2.83	3	0.419	
Year (1)	−0.346	1.032	0.113	1	0.737	0.707
Year (2)	−1.18	1.015	1.353	1	0.245	0.307
Year (3)	−1.34	1.054	1.617	1	0.204	0.262
Marital status (1)	−1.559	0.912	2.921	1	0.087	0.21
Parental status (1)	0.586	1.406	0.174	1	0.677	1.796
GPA			1.229	3	0.746	
GPA (1)	−0.705	1.502	0.22	1	0.639	0.494
GPA (2)	−0.775	0.762	1.035	1	0.309	0.461
GPA (3)	−0.546	0.688	0.63	1	0.427	0.579
PSS-10	0.05	0.043	1.336	1	0.248	1.051
Cyclothymic	0.287	0.099	8.38	1	0.004 **	1.332
Depressive	−0.093	0.106	0.776	1	0.378	0.911
Irritable	0.845	0.166	26.037	1	<0.0001 **	2.329
Hyperthymic	−0.173	0.149	1.339	1	0.247	0.841
Anxious	−0.146	0.179	0.665	1	0.415	0.864
Constant	−4.878	2.144	5.178	1	0.023	0.008

Sig.: Significance; Exp.: Expected; * *p* < 0.05; ** *p* < 0.001.
